# Construction and characterization of a reverse genetics system of bovine parainfluenza virus type 3c as a tool for rapid screening of antivirals *in vitro*

**DOI:** 10.3389/fvets.2024.1336663

**Published:** 2024-03-12

**Authors:** Yu Han, Kejia Lu, Riteng Zhang, Xi Wei, Hanwei Guo, Lina Tong, Xinglong Wang, Sa Xiao, Haijin Liu, Zengqi Yang

**Affiliations:** ^1^College of Veterinary Medicine, Northwest A&F University, Yangling, China; ^2^College of Agriculture and Animal Husbandry, Qinghai University, Xining, China

**Keywords:** bovine parainfluenza virus, reverse genetics system, virus rescue, antiviral drugs, ribavirin

## Abstract

Bovine parainfluenza virus type 3 (BPIV3) is a key pathogen associated with bovine respiratory disease complex (BRDC). However, its specific pathogenesis mechanisms have not been fully elucidated. Reverse genetics provides a useful method for understanding the pathogenic mechanism of BPIV3. To ensure the functionality of the rescue platforms, we first constructed a minigenome (MG) system of BPIV3 utilizing a 5-plasmid system in this investigation. Then, a full-length infection clone of BPIV3 was obtained from the SX-2021 strain, and different methods were employed to identify the rescued virus. Additionally, we recovered a recombinant BPIV3 using the reverse genetics system that could express enhanced green fluorescence protein (eGFP). Through the growth curve assays, the replicate capability of rBPIV3-SX-EGFP was found to be similar to that of the parental virus. Subsequently, the rBPIV3-SX-EGFP was used to determine the antiviral activity of ribavirin. The results showed that ribavirin had an anti-BPIV3 effect in MDBK cells. In conclusion, the successful development of a reverse genetic system for the SX-2021 strain establishes a foundation for future studies on BPIV3, including investigations into its pathogenic mechanism, gene function, and antiviral screening properties.

## 1 Introduction

BPIV3 is recognized as a significant pathogen contributing to BRDC, inducing a spectrum of mild-to-severe respiratory symptoms (1, 2). BPIV3 is a non-segmented, enveloped negative-strand RNA virus that is a member of the *Respirovirus* genus within the *Paramyxovirdae* family (2). In recent years, there has been an increasing number of BPIV3-induced respiratory illness outbreaks in cattle, resulting in substantial financial losses within the global livestock industry (3–5). BPIV3 has been classified into three genotypes, and the first report of BPIV3 genotype C as a new genotype was identified in China (6). Nevertheless, BPIV3c lacks a recognized therapy, and its pathogenic mechanism remains elusive.

To date, only limited studies on the reverse genetics of the BPIV3 genotype A have been reported (7, 8). Therefore, it is crucial to establish a reverse genetics system for BPIV3c to investigate pathogenic mechanisms and facilitate the development of a modified live virus vaccine. Mini-genome (MG) systems, also referred to as Mini replicon technology, are commonly utilized to explore the life cycle of viruses within the *Paramyxoviridae* family (9–11). Previously, reporter viruses have been effectively employed to examine the pathophysiology of *paramyxoviruses* and screen antiviral medicines with high sensitivity. Ribavirin, a broad-spectrum antiviral drug, is commonly used in antiviral tests (12).

In this study, we successfully developed the first reverse genetic system for BPIV3 genotype C using a 5-plasmid system. Furthermore, the rBPIV3-SX-EGFP was employed as a screening method for antivirals *in vitro*.

## 2 Materials and methods

### 2.1 Cells, virus, plasmids, and antibodies

MDBK and BHK-21 cells were cultured in Dulbecco’s modified Eagle’s medium (DMEM) (Gibco, Carlsbad, CA), supplemented with 10% fetal bovine serum (FBS) (Gibco, Carlsbad, CA), at 37°C with 5% CO_2_. The BPIV3 strain was isolated from a BPIV3-positive lung tissue sample, designated as BPIV3/SX/2021 (GenBank: ON804787). The pCMV and pCI-neo plasmids were used to construct the rBPIV3-SX cDNA clone and three helper plasmids, respectively. The pGAGGS-T7 plasmid, containing T7 RNA polymerase, was constructed in our laboratory. Polyclone antibodies against NP were prepared in our laboratory, and the specificity has been verified in our previous study (13). An anti-EGFP antibody (ab184601) was purchased from Abcam. HRP-conjugated Goat Anti-Mouse IgG was purchased from Invitrogen. FITC-labeled Goat Anti-Mouse IgG (H + L) was purchased from Beyotime.

### 2.2 Construction of plasmids

A set of primers was designed to construct an MG system for BPIV3 based on the conserved genomic regions of the viral 5′ untranslated region (5′ UTR), EGFP, and the viral 3′ UTR. The three PCR products were assembled by overlap and inserted in the antisense orientation between the T7 promoter and terminator sequences, resulting in a construct named pT7-MG.

**Figure 1:**
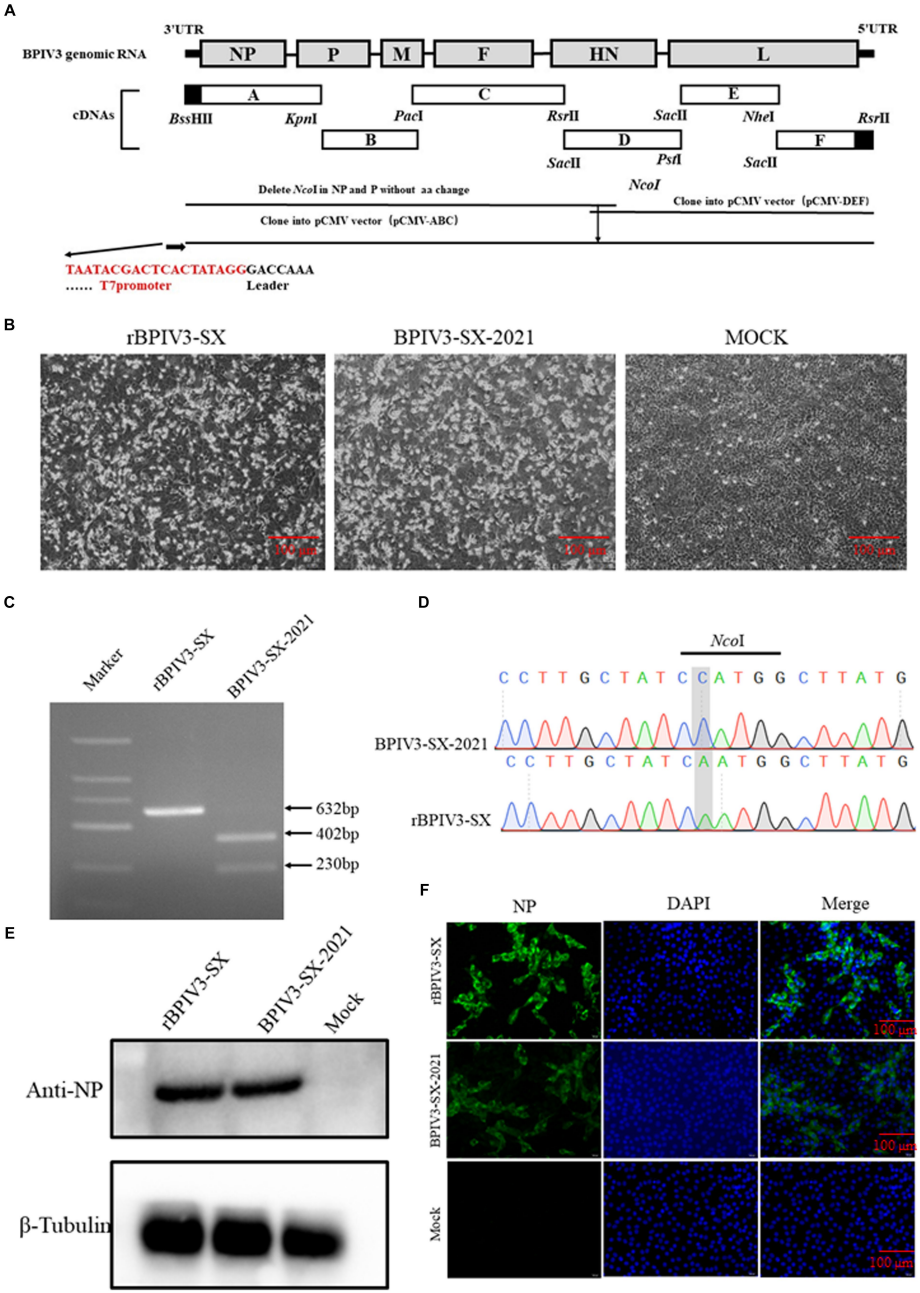
Identification of the rescued rBPIV3-SX virus assay. **(A)** Schematic representation showing the construction of pBPIV3-SX. **(B)** BPIV3-specific cytopathic effect (CPE) of the rescued virus. MDBK cell cultures were collected at 48 h after infection with the rescue and parental virus. **(C)** Identification of genetic marker in the rescued virus. The *Nco*I restriction enzyme site was mutated into the recombinant plasmid to create a genetic marker to distinguish the rescued virus from the parental virus. **(D)** The genetic marker mutation was identified by sequencing. **(E)** Expression of NP in rBPIV3-SX-infected cells. The MDBK cells infected with the rescue and parental virus were detected using Western blotting using anti-BPIV3 NP polyclonal antibody at 24 h. **(F)** Immunofluorescence analysis of the rescued virus. The MDBK cells were infected with the rescue and parental virus, and the infected cells were stained by anti-BPIV3 NP polyclonal antibody.

To construct a full-length cDNA clone of the SX-2021 strain, a set of primers was designed to amplify the viral RNA genome using reverse transcription-polymerase chain reaction (RT-PCR). The PCR products were sequenced by Sanger sequencing with three independent parallel tests. Six separate fragments (A to F) were assembled using unique restriction enzyme sites. A T7 promoter was inserted upstream of fragment A and three G residues preceding the T7 promoter sequence. Fragment F contained a 24-nucleotide (nt) partial HDV ribozyme (Supplementary Table S1). Additionally, a molecular marker for differentiating the rescued virus from the parental virus was created. The primers corresponding to fragments A and B were modified to eliminate the unnecessary *Nco*I site. The fragments of (A-C) and (D-F) were, respectively, inserted into the pCMV plasmid; pCMV (A-C) and pCMV (D-F) plasmids were digested by *Nco*I and *Rsr*II. These two fragments were then linked, and the full-length BPIV3 cDNA was assembled, named pBPIV3-SX (Figure 1A). The NP, P, and L ORFs were separately subcloned into the pCI-neo plasmid to construct the three helper plasmids, pCI-NP, pCI-P, and pCI-L.

To construct a rescue virus containing eGFP, a plasmid containing the full-length sequence of BPIV3 was used, with a fusion sequence of eGFP. The fusion sequence, flanked by *BssHII* and *PacI* sites, was subcloned into the pBPIV3-SX, resulting in the plasmid named pBPIV3-SX-EGFP.

### 2.3 Transfection and rescue of recombinant virus

BHK-21 cells were seeded into a 12-well plate and transfected with a plasmid of the minigenome or full-length cDNA clone, along with pCI-NP, pCI-P, pCI-L, and pCAGGS-T7 at a ratio of 0.5 μg: 0.5 μg: 0.1 μg: 0.05 μg: 0.35 μg, using TurboFect™ reagent (Invitrogen, Carlsbad, CA). Replication of this minigenome was observed at various time points under a fluorescent microscope. After 60 h post-transfection with the full-length cDNA clone, the cell supernatant from BHK-21 cells was harvested and transferred to MDBK cells. The rescue viruses were harvested between 48 and 60 h post-infection when significant CPE was observed. Additionally, the rescue viruses underwent 20 serial passages in MDBK cells, using a multiplicity of infection (MOI) of 0.1. The virus was adsorbed onto confluent cell monolayers in 60-mm cell plates for 1 h at 37°C with frequent rocking. Following adsorption, the inoculum was removed, the cells were washed with DMEM, 4 mL of maintenance media was added, and the plates were incubated at 37°*C. medium* from each well was harvested at 72 hpi and stored in aliquots at −80°C. Following each passage, the viral harvest was quantified by TCID_50_ assay on MDBK cells and diluted to produce an MOI of 0.1 for each subsequent passage.

### 2.4 Identification of recombinant viruses

To determine the molecular marker (C341 to A341 mutation in the *Nco*I site) in the rescued virus, rBPIV3/SX, RT-PCR was performed on the genomic region containing the specific mutation. For this purpose, viral RNA was extracted from passage 5 of the rBPIV3-SX virus using the TRIzol reagent (Takara) and reverse transcribed into cDNA using a StarScript II RT Kit (Genstar, Beijing, China). RT-PCR was employed to amplify the relevant viral genomic region using the primer pair, Nco-F/Nco-R (Supplementary Table S1). The PCR product was gel-purified, digested with *Nco*I, and the DNA sequenced to confirm the presence of the C341 to A341 mutation.

### 2.5 Western blot analysis

MDBK cells were seeded into a 12-well plate and were infected with BPIV3-SX-2021, rBPIV3-SX, rBPIV3-SX-EGFP, or mock-infected at an MOI of 0.1. Cell lysates were harvested using 160 μL/well lysis buffer at 24 hpi, and the cell lysate was denatured at 95°C for 10 min in a 5x loading buffer (Bio-Rad, Hercules, CA). Proteins were analyzed on a 10% SDS-PAGE and then transferred to a PVDF membrane. The membrane was blocked with 5% skim milk for 2 h at room temperature. To identify the expression of BPIV3 NP or eGFP, the membrane was incubated overnight at 4°C with primary antibodies. The membrane was then washed three times with TBST, followed by incubation with secondary antibodies at room temperature for 1 h. Target proteins were analyzed using BeyoECL Plus (Beyotime, Beijing, China).

### 2.6 Immunofluorescence assay

MDBK cells were seeded into a 24-well plate and infected with BPIV3-SX-2021, rBPIV3-SX, and rBPIV3-SX-EGFP at an MOI of 0.1 or mock-infected. At 24 hpi, cell monolayers were fixed with 4% paraformaldehyde at room temperature for 30 min and then blocked with PBS containing 1% bovine serum albumin (BSA) for 30 min and then permeabilized with 0.1% Triton X-100 at 5 min. Cell monolayers were incubated with pAb against NP for 1 h and then washed with PBS three times. Cell monolayers were further incubated for 1 h with a secondary antibody. After washing three times, the cells were analyzed under a fluorescent microscope (IX73; OLYMPUS, Tokyo, Japan).

### 2.7 TCID_50_ assay and virus growth kinetics

MDBK cells were seeded into a 6-well plate and infected with BPIV3-SX-2021, rBPIV3-SX, and rBPIV3-SX-EGFP at an MOI of 0.1. The plates were incubated at 37°C for 1 h, and the cell culture supernatant was removed and replaced with DMEM supplemented containing 2% FBS. Cell culture supernatants were harvested at 12, 24, 36, 48, 60, and 72 hpi, and the viral titers were measured using the TCID_50_ assay on MDBK cells.

### 2.8 Anti-rBPIV3-SX assays of ribavirin

To determine the concentration of ribavirin-treated effect on MDBK cell viability, a CCK-8 assay was employed (Solarbio, CA1210). MDBK cells were seeded into a 96-well plate, and the supernatants were supplemented with DMEM containing ribavirin at various dosages. After incubating the ribavirin-treated cells for 24 h, the CCK-8 solution (10 μL/well) was added to each well. The plate was examined using a microplate reader at 450 nm after 4 h of incubation.

MDBK cells were seeded into a 12-well plate to assess the antiviral activities of ribavirin at various doses. The cells were infected with rBPIV3-SX-EGFP at an MOI of 1 for 1 h at 37°C. The supernatants were replaced and added to DMEM containing ribavirin at various doses. A fluorescent microscope was used to examine ribavirin-treated cells after 24 h. The supernatants were harvested for the viral titer determination using the TCID_50_ assay of rBPIV3-SX-EGFP.

## 3 Results

### 3.1 Construction of a minigenome system of BPIV3

**Figure 2:**
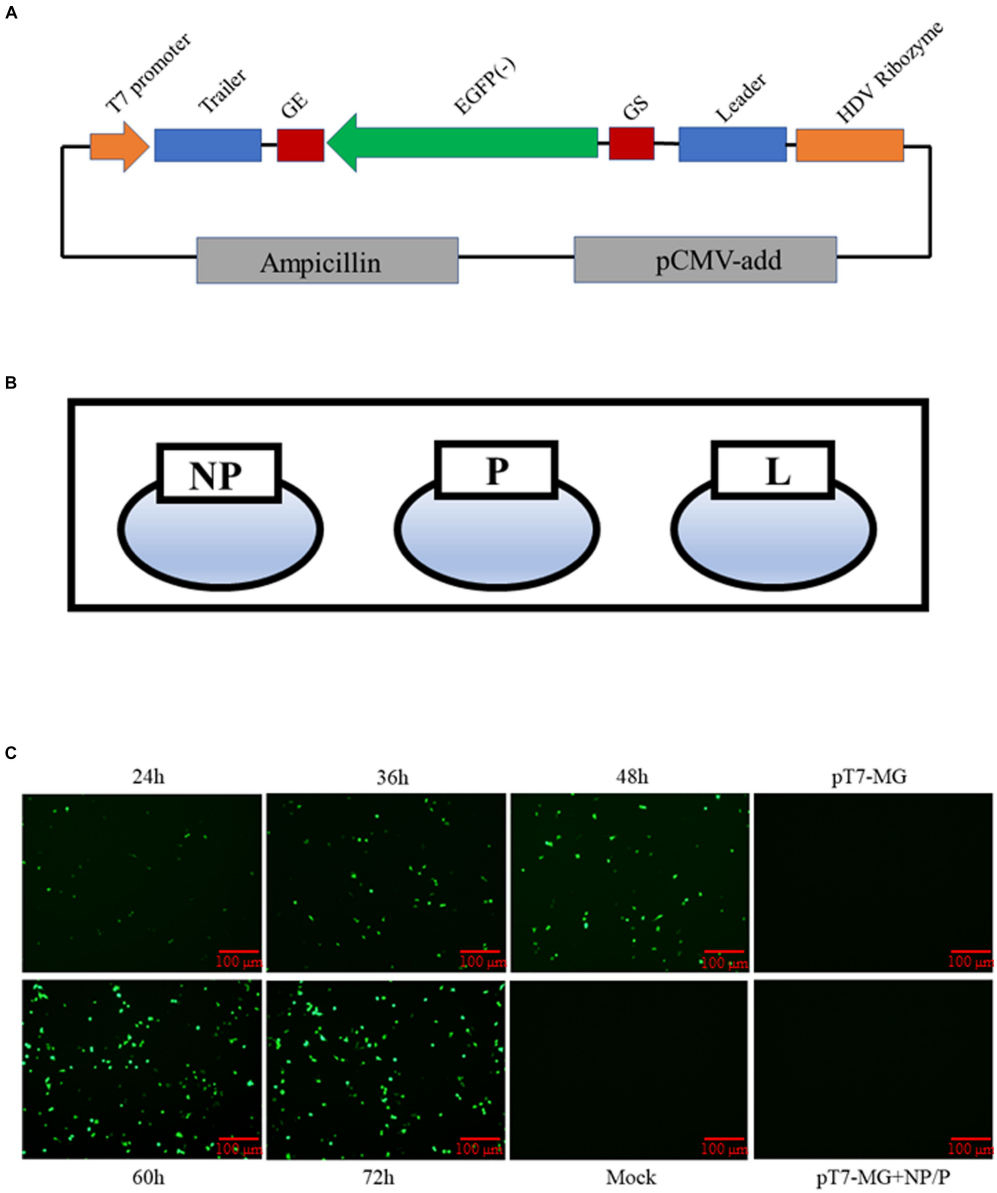
Construction of BPIV3 minigenome system. **(A)** Diagram showing the structure of the BPIV3 minigenome system. This reporter plasmid contains the eGFP reporter gene inserted between the leader and trailer regions and then flanked by the T7 promoter and HDV ribozyme. **(B)** Schematic map of the three helper plasmids for the rescue virus. **(C)** BHK-21 cells co-transfected with five plasmids at 24 h, 36 h, 48 h, 60 h, and 72 h. The expression levels of eGFP were determined under the fluorescence microscope.

In addition to reporter viruses, a minigenome system is a powerful tool for viral studies. Consequently, we constructed a minigenome system, with the 5′ and 3′ non-coding regions of the BPIV3 genome constituting the trailer and leader sequence. The genome structures of two genes were flanked by gene start (GS) and gene end (GE) sequences. The reporter plasmid contains the eGFP reporter gene inserted between the leader and trailer regions and is flanked by the T7 promoter and HDV ribozyme. The MG sequence of BPIV3 was schematically shown in Figure 2A. In addition to the MG plasmid, the three helper plasmids were used in the minigenome system (Figure 2B). The (pT7-MG) plasmid and pCAGGS-T7 plasmid, along with the three helper plasmids, were co-transfected into BHK-21 cells. The replication of this minigenome (MG) and eGFP activity was assessed at various time points. At 24 hpi, a few cells exhibited green fluorescence, with the proportion of such cells increasing over time. The eGFP activity increased substantially from 24 to 72 h post-transfection, indicating that our MG system replicated efficiently (Figure 2C).

### 3.2 Rescuing and identification of rBPIV3

To rescue the virus, the full-length infection clone plasmid of rBPIV3-SX was constructed and co-transfected into BHK-21 cells with the helper plasmids. After 72-h transfection, the supernatants from transfected cells were harvested and subsequently transferred onto the MDBK cells. Simultaneously, typical CPEs were observed in virus-infected cell monolayers at 48 h (Figure 1B). Total RNA was extracted from rBPIV3-SX or the parental virus, and the fragment containing the *Nco*I molecular marker in rBPIV3-SX was analyzed by RT-PCR using the primer *Nco*-F/R. PCR products were gel-purified and digested by *Nco*I. The parental virus produced a 402-bp and a 230-bp fragment following *Nco*I digestion, whereas the rescued virus could not achieve this (Figure 1C). Additionally, the PCR products were sequenced to confirm the presence of the *Nco*I site mutation (Figure 1D). The expression of NP protein in rBPIV3-SX infected cells was analyzed using the Western blot. The result showed the NP (69 kDa) protein was expressed in cells infected with the rBPIV3-SX (Figure 1E). BPIV3 NP expression was further assessed using a pAb against the NP protein by IFA. The virus-infected cell monolayer was visible in bright green fluorescence but not in the mock cells (Figure 1F).

### 3.3 Construction of infection clones containing a reporter gene

**Figure 3:**
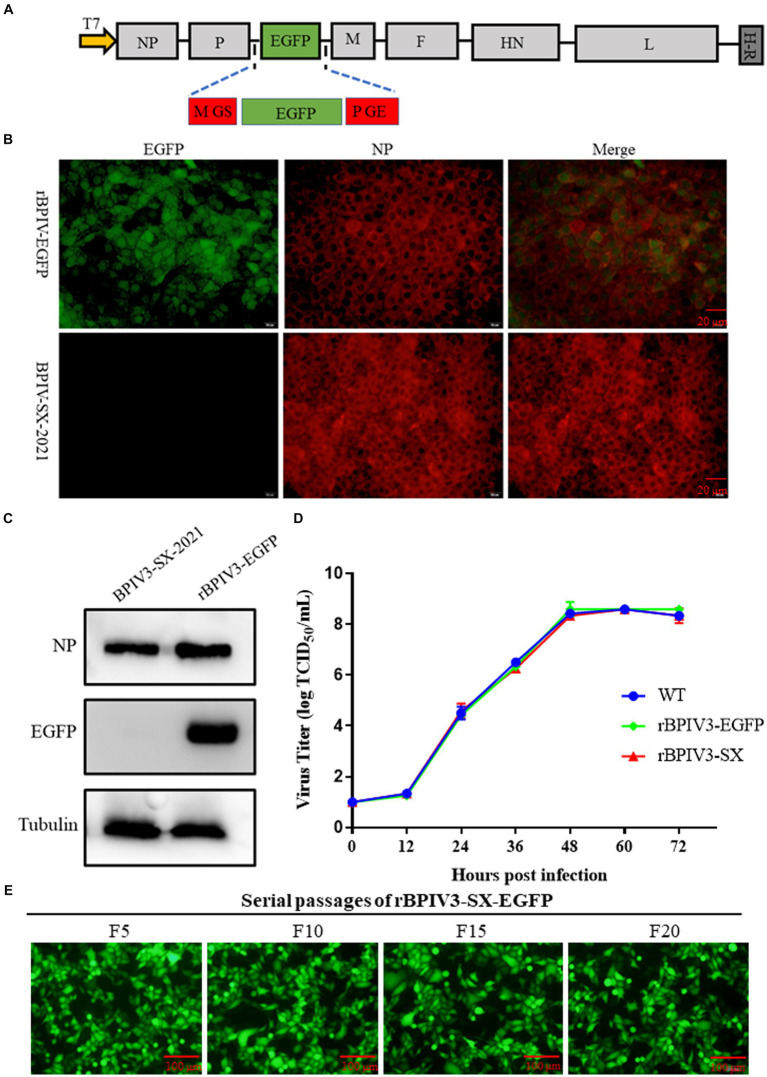
Construction, identification, and characterization of full-length BPIV3 reporter virus. **(A)** Diagram showing the structure of the full-length BPIV3 reporter virus. The H-R stands for HDV ribozyme. **(B)** Immunofluorescence analysis of the rescued virus. The MDBK cells were infected with rBPIV3-SX-EGFP and parental virus; the infected cells were stained by anti-BPIV3 NP polyclonal antibody. **(C)** Expression of NP and eGFP in rBPIV3-SX-EGFP-infected cells. The MDBK cells infected with rBPIV3-SX-EGFP and parental virus were detected using Western blotting using anti-BPIV3 NP polyclonal antibody and anti-EGFP monoclonal antibody at 24 h. **(D)** The growth kinetics of the rescued viruses. MDBK cells in six-well plates were infected with rBPIV3-SX, rBPIV3-SX-EGFP, and parental virus. The supernatant was harvested at 12, 24, 36, 48, 60, and 72 h and titrated on MDBK cells. **(E)** Serial passages of rBPIV3-SX-EGFP. The MDBK cells were infected with different passages of rBPIV3-SX-EGFP, and the expression of eGFP was determined under the fluorescence microscope.

Furthermore, we generated a full-length infectious clone of the BPIV3 containing an eGFP reporter. The P-M intergenic site has been demonstrated to be preferable for the optimal expression of foreign proteins (14). Using the rBPIV3 (SX-2021) cDNA clone as the backbone of the rBPIV3-SX-EGFP cDNA clone, an EGFP reporter gene was inserted between BPIV3 P and M genes of the rBPIV3-SX cDNA clone using overlapping technology (Figure 3A). To rescue the rBPIV3-SX-EGFP, we co-transfected the full-length plasmid and the helper plasmids into BHK-21 cells. As depicted in Figure 3B, to characterize the recombinant virus, the viral protein NP was confirmed using immunofluorescent microscopy, and the eGFP and NP proteins were expressed in rBPIV3-SX-EGFP virus-infected cells. To further determine the expression of NP and eGFP proteins using the Western blot, the results showed that the NP (69 kDa) and eGFP (27 kDa) proteins were expressed in cells infected with rBPIV3-SX-EGFP. However, only the NP protein was expressed in cells infected with the parental virus (Figure 3C). To assess the impact of eGFP expression on viral replication, the growth kinetics of rBPIV3-SX-EGFP were compared to those of rBPIV3-SX and the parental virus. The results have shown that all the viruses displayed a similar growth behavior during 72 h (Figure 3D). To evaluate the genetic stability of the recombinant virus, rBPIV3-SX-EGFP underwent 20 serial passages in MDBK cells. As shown in Figure 3E, eGFP was consistently expressed at a high level during passages.

### 3.4 Reporter virus for antiviral testing

**Figure 4:**
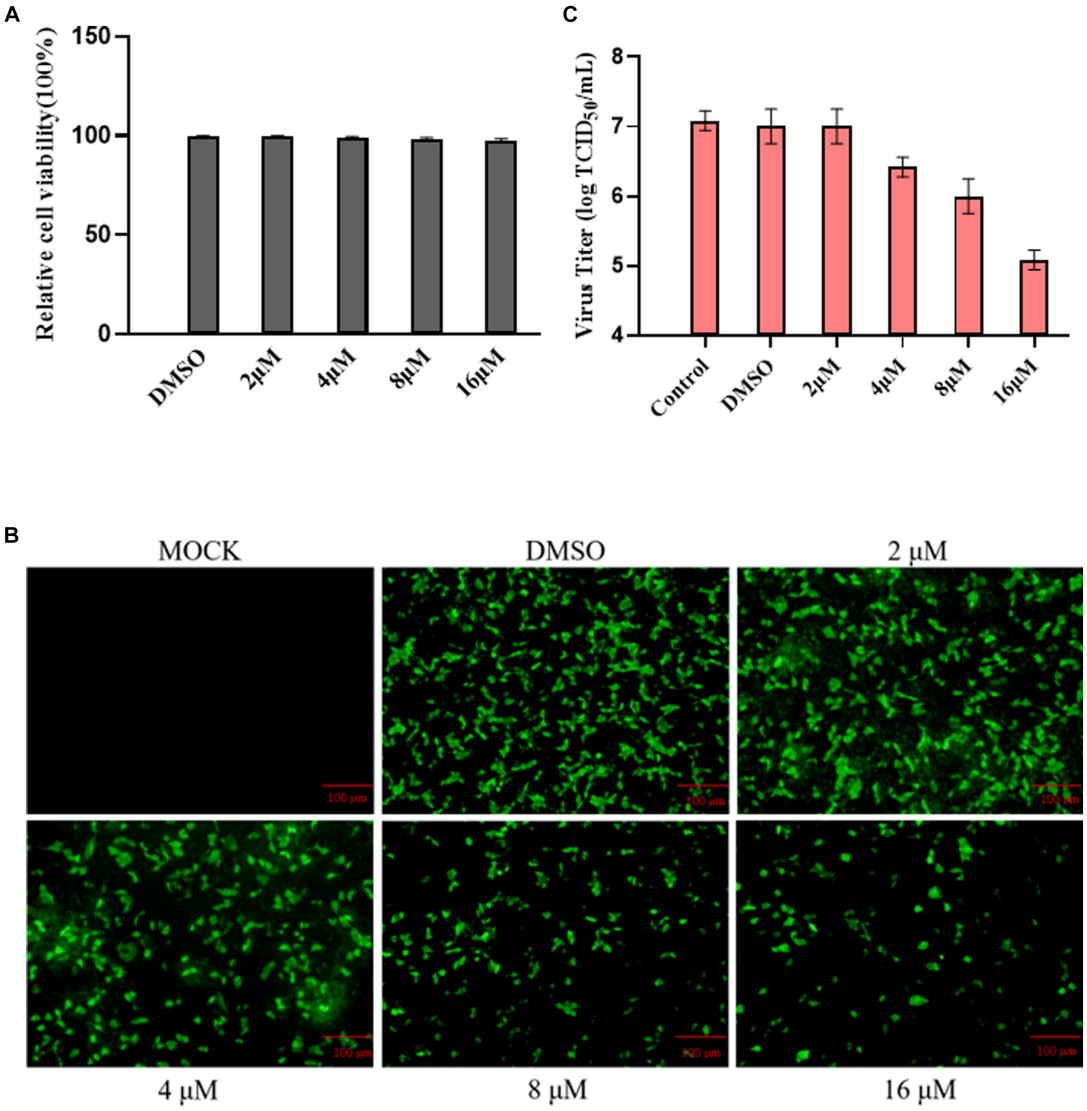
Ribavirin inhibitions of rescued rBPIV3-SX-EGFP replication. **(A)** The cytotoxic effect of ribavirin treatment on MDBK cells. The cells were treated with 0, 2, 4, 8, or 16 μM. Relative cell viability was determined using the CCK-8 assay. **(B)** Profiles of rBPIV3-SX-EGFP-expressed eGFP at 24 h after treatments separately with ribavirin at different concentrations. **(C)** The viral titer was detected with different concentrations of ribavirin treatment using the Reed–Muench assay.

Using the Cell Counting Kit 8 assay, we assessed the impact of ribavirin on MDBK cell viability. The results showed that ribavirin treatment did not affect the viability of the cells (Figure 4A). Ribavirin-treated cell monolayers were monitored at 24 hpi in a randomly selected field-of-view fluorescence microscope to assess the declining trend in eGFP expression. The results showed that the fraction of green cells gradually decreased with increasing drug concentration in the group treated with ribavirin (Figure 4B). At 24 hpi, all ribavirin-treated cell cultures were titrated using the TCID_50_ assay, and the viral titer steadily dropped with increasing ribavirin concentration, as observed under the fluorescence microscope (Figure 4C).

## 4 Discussion

BPIV3 infection primarily induces respiratory symptoms in both young and adult cattle, such as cough, fever, and nasal discharge, particularly in feedlot cattle (15, 16). Genotype C of BPIV3 as a potentially new genotype was isolated and identified in China in 2008 (6). Subsequently, BPIV3c has been identified in major cattle-producing countries, including the United States, Australia, Japan, and Korea. Its presence has led to significant economic losses in the global livestock industry (4, 5, 17).

Previous phylogenetic studies have revealed significant genetic diversity among different genotypes of BPIV3, with potential geographical and tempo ral ranges (6). Therefore, the construction of the reverse genetic systems for different viral strains could be useful for assessing the evolutionary and ecological assessments of BPIV3. The reverse genetics system for BPIV3 dates back to as early as 2000 when it was established as an RNA virus vector for expressing and distributing foreign antigens, contributing to disease prevention in humans (7). In this study, we first successfully built a reverse genetic system for the BPIV3c strain, SX-2021. To construct the reverse genetic system, we first developed a minigenome system (MG) to test the utility of the helper plasmids. Furthermore, the MG system is a powerful tool commonly utilized to study the life cycle of members of the *Paramyxoviridae* family. Moreover, the ratio of the helper plasmids has been shown to influence the efficiency of MG reporter gene expression (18). However, the MG system, being shorter than the full-length genome, has inherent limitations that need to be acknowledged. Therefore, rescuing a new recombinant virus has provided significant advantages for further studying the function of viral genes. To rescue the recombinant virus, we constructed a stable full-length cDNA clone carrying a genetic marker. This clone was then co-transfected with the infection clone into BHK-21 cells along with the helper plasmids. The rescued rBPIV3-SX and parental BPIV3 exhibited identical characteristics in the Western blot and immunofluorescence assays. To determine the genetic marker stability of the rescued rBPIV3-SX, we employed restriction enzyme analysis and Sanger sequencing.

A pivotal application of the reverse genetic system involves utilizing the viral backbone for expressing foreign genes (18). In a previous study with the BN-1 strain, a recombinant virus containing the eGFP reporter gene was constructed and inserted between the N and P genes (8). However, the reporter virus was only used to examine the tropism and pathogenesis of BPIV3 in hamsters. Previous studies on other paramyxoviruses have indicated that the expression of foreign proteins, with varying insertion sizes, could impact virus replication (19–21). In this study, we inserted reporter protein gene sequences between the P and M proteins in BPIV3, immediately fused with gene-start (GS) and gene-end (GE) sequences (14, 22). Upon comparing the replication of rBPIV3-SX-EGFP with wild-type parental BPIV3, our results demonstrated that rBPIV3-SX-EGFP exhibited similar growth kinetics to the parental virus. Additionally, the utilization of virus expression reporter genes serves as a powerful tool for screening and testing antiviral drugs (23–25). Ribavirin is a purine nucleoside derivative with broad-spectrum antiviral activity. In this study, we employed rBPIV3-SX-EGFP for drug cytotoxicity and viral infection tests to evaluate its potential in discovering novel antiviral drugs and conducting high-throughput drug screening for anti-BPIV3 drugs. These findings suggest that the rBPIV3-SX-EGFP holds promise for discovering novel antiviral drugs and conducting high-throughput drug screening for anti-BPIV3 drugs.

In summary, our success in developing both a minigenome system and a full-length genome for an emerging BPIV3c represents a significant achievement. Additionally, we successfully rescued a recombinant virus containing an eGFP reporter gene. This tool will be instrumental in conducting tests for antiviral drugs. We believe that the minigenome system, full-length genome, and the recombinant virus with an eGFP reporter gene can collectively contribute to further investigations into the biology, tissue tropism, and transmission dynamics of BPIV3.

## Data availability statement

The datasets presented in this study can be found in online repositories. The names of the repository/repositories and accession number(s) can be found in the article/ Supplementary material.

## Author contributions

YH: Writing – original draft. KL: Investigation, Writing – original draft. RZ: Software, Writing – original draft. XW: Validation, Writing – original draft. HG: Resources, Writing – original draft. LT: Investigation, Writing – original draft. XLW: Resources, Writing – original draft. SX: Data curation, Writing – original draft. HL: Formal analysis, Writing – original draft. ZY: Writing – review & editing.

## Supplementary material

The Supplementary material for this article can be found at: https://www.frontiersin.org/articles/10.3389/fvets.2024.1336663/full#supplementary-material
