# Supplementary material for: Construction and characterization of a reverse genetics system of bovine parainfluenza virus type 3c as a tool for rapid screening of antivirals in vitro
Source: Front Vet Sci. 2024 Mar 12;11:1336663. doi: 10.3389/fvets.2024.1336663 (PMC10967227; doi:10.3389/fvets.2024.1336663)
Supplement: Supplementary file 1 [file Table_1.DOCX]

Supplementary Material

# Supplementary Figures and Tables

## 1.1 Supplementary Tables

**Table S1.** Primer pairs were used in this study.

| Primers | Sequences（5’-3’） |
| --- | --- |
| T7-trailer-F-BsshII | TGAGCGCGCGTAATACGACTCACTATAGGGACCAAACAAGAGAAAAACTCTGTTTGGTA |
| trailer-R | ATGGACGAGCTGTACAAGTAGccgTGTATAAATCTAGTAAATAGTGTACTTAATCCCCAT |
| GFP-F | -CTACTTGTACAGCTCGTCCATGCCGA |
| GFP-R | -ATGGTGAGCAAGGGCGAGGAGCTGT |
| leader-F | TCCTCGCCCTTGCTCACCATGATGACAATTTAAGGATTTCTTCT |
| leader-R-Rsr II | GGTCGGACCGCGAGGAGGTGGAGATGCCATGCCGACCCACCAAACAAGAGGAGAGAATTGTTTGGAAATA |
| SA- F | AGGCGCGCGTAATACGACTCACTATAGGGACCAAACAAGAGGAGAGAATTGTTTGG |
| SA-R | GGTGGTACCTTGACTGGAACATTTTCAAGTT |
| SB-F | CAAGGTACCAAGAAGTGATGCCATAC |
| SB-R | GGTTAATTAATCCAAGATGGACCATAAAGTTT |
| SC-F | CCTTAATTAAACGGAAAGTAGGTCGAATGTAT |
| SC-R | GGTCCGGACCGCCATGGGCTGTAGGAGGCCT |
| SD-F | CCGCGGTACAGCCCATGGTTTTCAAATA |
| SD-R | AGTTGAATAACATATCCTGCAGTGCTCAGA |
| SE-F | CCGCGGGGTCTGAGCACTGCAGGATATGTTATTC |
| SE-R | TAACATGCTAGCATAGCACCTGCT |
| SF-F | CCGCGGGAGCAGGTGCTATGCTAGCATGTTA |
| SF-R | GGTCCGGACCGCGAGGAGGTGGAGATGCCATGCCGACCCACCAAACAAGAGAAAAACTCTGTT |
| SA-NcoI-F | AGTCTCCTTGCTATCaATGGCTTATGCTAA |
| SA-NcoI-R | TTAGCATAAGCCATtGATAGCAAGGAGACT |
| SB-NcoI-F | CCTGAACTGTACCCgTGGTCAAGCAGATTA |
| SB-NcoI-R | TAATCTGCTTGACCAcGGGTACAGTTCAGG |
| NCO-F | ATGTTGAGTCTGTTTGATACATTCA |
| NCO-R | CTTAATACCAGACTGGATTTGACTG |
| PCI-NP-F | CGGCTAGCATGTTGAGTCTGTTTGATACAT |
| PCI-NP-R | TTGCGGCCGCTTAGTTACTTCCGAATGCGC |
| PCI-P-F | CGGCTAGCATGGAAGACAATGTTCAAAACA |
| PCI-P-R | TTGCGGCCGCCTATTGGGAGCTGATGTCTT |
| PCI-L-F | CCCTCGAGATGGACACCGAATTCAGCGGTG |
| PCI-L-R | TTGCGGCCGCTTAATCAATATCAAATTCAT |
| EGFP-1F | GCCAATCAGTCCCTCGACAAAccgccaccATGGTGAGCAAGGGCGAGGAG |
| EGFP-1R | CTCCTCGCCCTTGCTCACCATggtggcggTTTGTCGAGGGACTGATTGGC |
| EGFP-2F | ATGGACGAGCTGTACAAGTAGACAGCCAAATGACAATCACC |
| EGFP-2R | GGTGATTGTCATTTGGCTGTCTACTTGTACAGCTCGTCCAT |
